# Addressing sex and gender to improve asthma management

**DOI:** 10.1038/s41533-022-00306-7

**Published:** 2022-12-20

**Authors:** Louis-Philippe Boulet, Kim L. Lavoie, Chantal Raherison-Semjen, Alan Kaplan, Dave Singh, Christine R. Jenkins

**Affiliations:** 1 Québec Heart and Lung Institute, Laval University, Québec City, QC Canada; 2 Department of Psychology, University of Quebec at Montreal (UQAM) and Montreal Behavioural Medicine Centre, CIUSSS-NIM, Hopital du Sacre-Coeur de Montreal, Montreal, QC Canada; 3 Centre Hospitalier Universitaire (CHU) de Guadeloupe, Guadeloupe, France; 4 INSERM U1219, EpiCene Team, University of Bordeaux, Bordeaux, France; 5 Department of Family and Community Medicine, University of Toronto, Toronto, ON Canada; 6 Division of Infection, Immunity and Respiratory Medicine, University of Manchester, Manchester University NHS Foundation Trust, Manchester, UK; 7 The George Institute for Global Health, Sydney, NSW Australia

**Keywords:** Asthma, Respiratory signs and symptoms

## Abstract

Sex (whether one is ‘male’ or ‘female’, based on biological characteristics) and gender (defined by socially constructed roles and behaviors) influence asthma diagnosis and management. For example, women generally report more severe asthma symptoms than men; men and women are exposed to different asthma-causing triggers; men tend to be more physically active than women. Furthermore, implicit, often unintended gender bias by healthcare professionals (HCPs) is widespread, and may result in delayed asthma diagnosis, which can be greater in women than men. The sex and gender of the HCP can also impact asthma management. Pregnancy, menstruation, and menopause can all affect asthma in several ways and may be associated with poor asthma control. This review provides guidance for considering sex- and gender-associated impacts on asthma diagnosis and management and offers possible approaches to support HCPs in providing personalized asthma care for all patients, regardless of their sex or gender.

## Introduction

Globally, ~300 million people live with asthma^1^. There are well-established sex and gender differences in the prevalence of asthma: more boys than girls suffer from asthma pre-puberty, while post-puberty, the prevalence of asthma is higher in women than men^2^. Furthermore, women are more likely to have severe asthma, comorbidities, worse quality of life, and a higher rate of exacerbations, hospitalizations, and mortality compared with men^1,3^. These differences have been attributed to sex-specific physiological differences (e.g., sex hormones)^2^, but may also be driven by gender-specific sociocultural and behavioral differences (e.g., gender roles/occupations, symptom perception)^4,5^.

Importantly, ‘sex’ and ‘gender’ are not always clearly defined in scientific literature and are often used interchangeably and/or incorrectly^6^. Sex refers to the biological and physiological characteristics of females and males (e.g., chromosomes, hormones, and reproductive organs), while gender is a sociocultural construct that refers to the identities, characteristics, roles, and behaviors of men, women, boys, and girls and gender-diverse people^6^. Therefore, gender characteristics can vary between societies and may change over time.

In a previous review^7^, we outlined current evidence for sex- and gender-related differences that influence asthma pathogenesis, clinical course, severity, symptoms, and management. The aim of this narrative review is to provide guidance for healthcare professionals (HCPs) to consider sex- and gender-associated differences in asthma diagnosis and management when deciding the best course of action for their patients. These suggestions are based on the authors’ assessment of current evidence and are outlined in each section as “author guidance”.

There are limited studies on gender-diverse people with asthma, and many studies have been carried out using ‘traditional’ sex and gender definitions. As such, we have used the terminology of ‘men’ and ‘women’ throughout this review.

## Gender differences in patient health behaviors

### Patient reporting of symptoms

Patient reporting of symptoms may influence the way symptoms are interpreted by HCPs, and, therefore, how the patient’s asthma is managed. Evidence suggests that women perceive their asthma as more symptomatic than men and report more frequent, severe, and bothersome symptoms, even if the severity and level of asthma control are similar^4^. Compared with men, women also report poorer quality of life and greater symptom impact, including more limitations on sports, social activities, sleep, and day-to-day activities^8^. It may be for these reasons that women are more likely than men to report their symptoms to a HCP^8^. Men may also understate their symptoms and be reluctant to seek HCP support due to societal expectations that men should not complain about their health^9^.

Author guidance:

- Recognize that men and women may present with different symptom profiles and that gender can affect how and when patients report their symptoms.
- Confirm diagnosis with spirometry^10^, and use validated measurements of asthma control e.g., asthma control questionnaire or asthma control test, which help to objectively assess the severity of patients’ symptoms and response to treatment^10^.
- Discussion guides that help patients understand their asthma may prompt conversations to gain insight into their symptoms and daily life limitations so that appropriate support can be offered by HCPs and asthma educators^11,12^.

### Triggers and long-term exposures

**Fig. 1:**
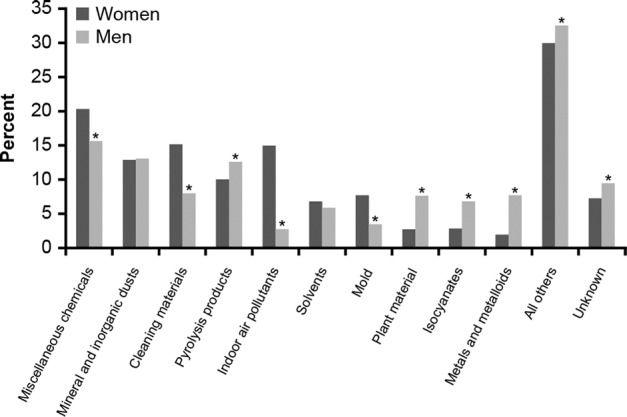
Major categories for reported exposures associated with work-related asthma by gender. Categories defined by the Association of Occupational and Environmental Clinics (AOEC), September 2012^5^; *p-value for gender differences <0.05. Percentages are based on the number of females (*n* = 4973) and males (*n* = 3264). Adapted from White et al.^5^. Reproduced with permission from Taylor & Francis © 2014. www.tandfonline.com.

While certain triggers, such as air pollution, are likely to be similar for both genders, women and men may be exposed to different triggers and asthma-causing substances due to their gender roles and occupations (Fig. 1)^5^. Globally, women are more likely to be exposed to cleaning chemicals and biomass fuels, while men are more likely to be exposed to pyrolysis products, plant-based materials, isocyanates, metals, and metalloids (all of which are associated with an increased risk of asthma and respiratory disease [Fig. 1])^5,13–17^. However, traditional gender roles are evolving, and it is important to explore the potential exposures faced by men and women in occupations that were once strongly aligned with a particular gender. Changing roles at work and home may result in the reduction of gender differences in exposure to asthma-exacerbating triggers.

On average, more men than women smoke^18^; however, it appears that women are more susceptible than men to smoking-related asthma symptoms^19^. Women who smoke are less likely than men to quit successfully as they are more susceptible to tobacco addiction: nicotine is metabolized faster in women than in men, resulting in a need for a higher level of nicotine to produce pleasurable feelings^20^. There may also be concerns for women surrounding cessation-related weight gain^21^.

**Fig. 2:**
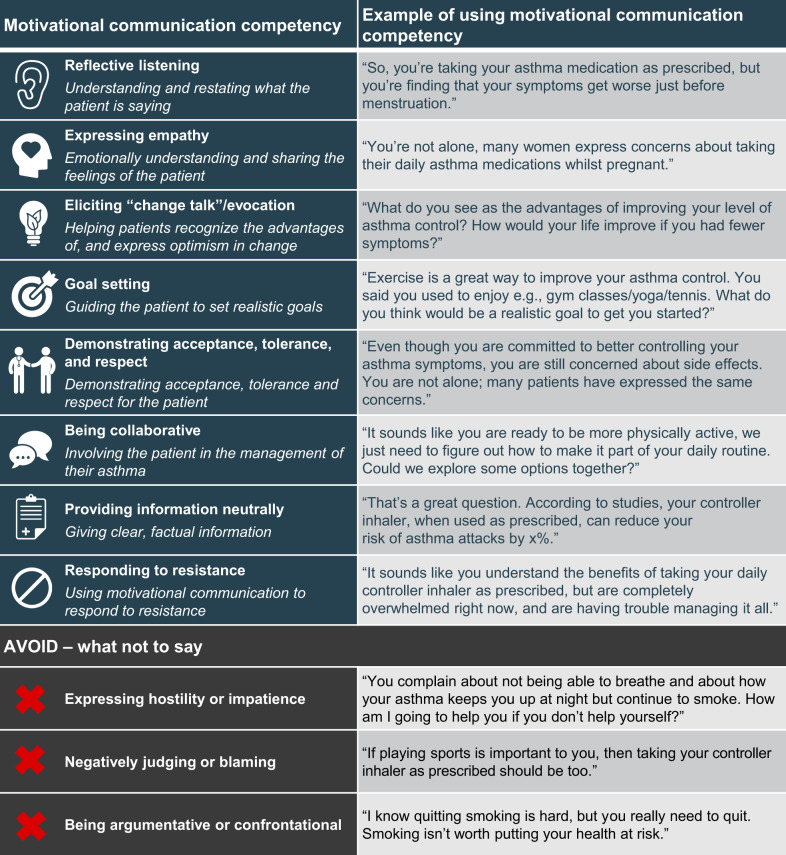
Motivational communication competencies, definitions, and examples. Adapted from Gosselin Boucher et al.^12^ under Creative Commons Attribution license CC-BY.

Author guidance:

- Be aware that, generally, men and women are exposed to different occupational and domestic triggers that may affect their asthma.
- Explore patient’s occupation, lifestyle and history to identify possible exposure to triggers. Discuss personal behaviors, workplace strategies, and explore protective measures to minimize exposure. If asthma symptoms persist or worsen, explore possible lifestyle and occupation changes.
- Although smoking cessation may be more difficult in women than men, due to factors such as susceptibility to tobacco addiction and weight-gain concerns, it should be encouraged regardless of patient sex/gender. Men and women often have different reasons for smoking or not quitting, and motivational communication (Fig. 2^12^) could be used to understand these reasons and tailor cessation advice.

### Physical activity and diet

Obesity and low levels of physical activity are associated with an increased risk of asthma symptoms^22^. Compared with women and girls, men and boys are more likely to participate in regular physical activity^23,24^ and are less likely to be obese^25^. However, men may have poorer diets than women; a large study that examined the relationship between gender, sexuality, and diet, reported that “very gender conforming males” (males with more ‘male’ personality traits) had unhealthier diets than the other groups examined^26^. It is known that a high intake of fruits and vegetables has anti-inflammatory properties that may reduce asthma risk and improve asthma control^27^, although more studies are needed on the relationship between diet and asthma outcomes.

Interventions that promote physical activity and healthy eating have been shown to improve asthma outcomes in both men and women^28^. It is important to note that men and women may have different goals and motivations for physical activity. Men have been shown to be motivated by competition, maintaining health, and enhancing body shape, whereas women are more motivated by emotional support and social aspects, as well as attaining well-being and a positive body image^29^.

Author guidance:

- Promote initiatives to educate all patients on the benefits of weight control, physical activity, and healthy eating on asthma control. Guide patients towards physical activities that they find enjoyable and beneficial.

### Adherence to asthma medication regimen

Taking medications correctly is important for asthma control. A Swedish study reported that, overall, men and women displayed similar levels of intentional nonadherence, but men with certain self-reported personality traits (e.g., agreeableness and conscientiousness) were more likely to adhere to their medication than men with more self-reported neurotic-type personality traits (e.g., vulnerability and self-consciousness)^30^; there was no association between personality traits and medication adherence in women^31^.

Women may be more likely to use their inhalation device incorrectly, which impacts asthma control^32,33^. Moreover, many patients rely on short-acting beta-agonists (SABAs) to treat their immediate asthma symptoms rather than taking daily maintenance medications that decrease the risk of exacerbations^10,34^. A Canadian study in patients ≥66 years old reported that women filled fewer prescriptions for maintenance inhalers and more prescriptions for reliever inhalers than men, suggesting that the women may also have had poorer asthma control^31^. As some people may not take their maintenance inhalers as prescribed and instead depend on reliever medication, the Global Initiative for Asthma (GINA) 2022 report recommends (in Track 1) low-dose inhaled corticosteroid (ICS)-formoterol as needed for mild asthma, or low-to-high dose ICS-formoterol as maintenance for more severe asthma (with low-dose ICS-formoterol as needed as a reliever). A second treatment track (Track 2) is possible, but not preferred^10^. It is important to consider that communities with limited resources can be found across low-, middle- and high-income countries; for these patients, Track 1 may not be affordable, and so Track 2 may be chosen while checking for adherence to treatment^10^.

Author guidance:

- Help patients to understand that asthma is a chronic condition that can be controlled by taking asthma medication as prescribed and using inhalers correctly, in combination with self-management (e.g., reducing allergen exposure, engaging in a healthy lifestyle).
- Educate patients on self-management to help them identify symptom worsening. Self-management education that includes a written action plan, regular review and symptom monitoring, reduces unscheduled visits, hospitalization and time lost from school or work^10^. A plan to explain when and how to increase medication(s) and when to seek HCP input may help patients gain greater control of their asthma, increase confidence about getting active and reduce asthma exacerbations^35^.
- Assess the frequency of SABA use. In patients who are dependent on SABAs and/or are avoiding regular maintenance medication, ICS-formoterol can be used “on demand” in mild asthma (anti-inflammatory reliever therapy), or regularly twice-daily plus on demand if asthma is more severe^10^. For severe asthma, treatment regimens may be simplified (if considered appropriate and the patient is adherent) with a single once-daily inhaler with a long-acting (24 h) ICS/beta-agonist association, plus long-acting muscarinic antagonist if required^10,36^. Alternatively, add-on biologic therapy can be considered if these therapies are not sufficient to treat persistent symptoms, following a specialized consultation and review of potential care gaps^10^.
- Assess asthma phenotype to tailor treatment and optimize asthma control.
- Check inhaler technique regularly. Poor inhaler technique is common, and contributes to poor asthma control, along with overuse of SABAs and short-course oral corticosteroids (OCS)^10,37^.

## Gender bias in HCP behavior

Implicit gender bias by HCPs is widespread and may affect the diagnosis, management, and health outcomes of diseases/conditions^38^. A 2019 analysis in Denmark found that in 72% of cases (of various diseases, including respiratory diseases), the median time span from symptom onset to diagnosis was longer in women than in men^39^. Moreover, in patients with chronic pain, men are often viewed as ‘brave’ while women are viewed as ‘emotional’ and ‘complaining’^40^. In a recent survey of women in England, 84% said they felt they were not listened to by HCPs^41^. This implicit bias may prevent women from receiving adequate asthma care^40^.

Asthma with comorbid anxiety is more prevalent in women than men^42,43^. As anxiety and stress can lead to HCPs taking patients’ symptoms less seriously^40^, it is possible that people with comorbid anxiety (women in particular) may be more likely to be misdiagnosed and/or receive sub-optimal asthma management^44^. In a 2006 study of patients with chronic obstructive pulmonary disease (COPD), a hypothetical case study was given to primary care physicians (PCPs), with half told the patient was a woman, and half told the same patient was a man. Results showed that COPD was more likely to be diagnosed in men than women, although this gender bias no longer appeared once the physicians were shown the patients’ spirometry results^45^. It is possible that assumptions that women are less likely to smoke and more likely to manifest anxiety as respiratory complaints may have played a role.

Spirometry is a vital tool to help confirm an asthma diagnosis but is under-utilized in diagnosing asthma in primary care^10,46^; this has been exacerbated by infection-control precautions during the COVID-19 pandemic. Under-utilization of spirometry may be more predominant in women: a recent study in patients ≥66 years old with asthma, reported that women experienced significantly lower rates of spirometry than men^31^. The reasons for this are unclear, but the authors suggest it could be related to either provider and/or patient behaviors. Nevertheless, the study highlights the importance of basing asthma diagnoses on objective measures like spirometry, peak flow, and fractional exhaled nitric oxide (FeNO) tests, as women in the study also had higher rates of Emergency Department (ED) visits than men. Recent studies of spirometry data from transgender and gender non-binary patients have also demonstrated that, although spirometry reference values should be based on birth sex and not gender^47^, until very recently, there has been a lack of guidance and hence inconsistent use of male and female reference ranges^48^. HCPs are often unsure whether reference ranges for birth sex or gender should be used, especially if patients feel discriminated against if birth sex is used^49^. This uncertainty may result in systemic or unconscious provider biases^48^ and may lead to misdiagnosis and inappropriate treatment^50^.

The gender of the HCP may also influence disease management. In a study of women general practice nurses, the nurses provided significantly more comprehensive information to women but discussed disease management more with men^51^. Furthermore, a 2020 study showed that female HCPs consulting with male patients discussed preventative interventions and lifestyle modification more often than any other patient–HCP gender combination^52^. This could be significant as it is probable that asthma educators are more likely to be women than men (as frontline HCPs are more likely to be women).

Author guidance:

- Be aware of implicit gender bias, and the impact this has on asthma diagnosis, time to diagnosis, choice and interpretation of tests, and management. Gender bias training and self-assessment tools are recommended to gain these insights. HCPs can assess their gender bias using the Harvard University self-assessment tool^53^.
- Eliminating gender bias should help in treating patients as individuals. Health behavior concerns should be addressed equally in men and women; for example, although more women than men may be physically inactive, it is important to still ask men about their physical activity as well as women.
- Be aware of the importance of spirometry and its utilization to avoid both under- and over-treatment of asthma in men and women. Bronchial provocation (e.g., with methacholine), may also be used if a diagnosis cannot be reached^10^.
- As sex is one of the predictive criteria for spirometry, consider what reference value ranges should be used for transgender patients. The 2019 American Thoracic Society and European Respiratory Society guidelines specify that patients should be informed that “birth sex and not gender is the determinant of predicted lung size” and using non-birth sex to calculate predicted spirometry values may lead to misdiagnosis and inappropriate treatment^47^.

## HCP‒patient relationship

Good HCP–patient communication is vital, and time spent with patients exploring their concerns, encouraging health behavior changes, and supporting self-management can improve medication adherence and asthma control^11^. Motivational communication is a form of patient-centered behavior-change counseling that focuses on enhancing internal motivation to engage in appropriate self-management behaviors^11,12,54^. There is evidence that the impact of motivational communication can depend on the gender of the patient and of the HCP^51^.

The GINA 2022 report recommends that patients’ own healthcare goals and treatment preferences are incorporated into their asthma management plan^10^. However, it is important to recognize that women and men may have different healthcare goals. A recent study reported that men were more likely than women to focus on disease-specific goals (e.g., asthma control and medication reduction) rather than function-related (e.g., social, emotional) or knowledge-related (e.g., asthma education) goals. Better asthma control was achieved when patients (regardless of sex/gender) focused on disease-specific goals^55^.

Author guidance:

- Consider undertaking evidence-based training on motivational communication (Fig. 2)^12^. As the impact of motivational communication can depend on the gender of the patient and the HCP^51^, training undertaken should try to address this discrepancy.
- Prioritize spending time with patients at the start of the relationship, as this can save time spent in the long run^11^. Specialist asthma educators, pharmacists, school nurses and other HCPs can also support PCPs in educating/supporting patients with asthma^56,57^.
- Perform regular asthma reviews with all patients; discuss the patient’s own healthcare goals and encourage them to focus on disease-specific goals.
- Implement the GINA cycle of care for all patients^10^.

## Management of asthma during pregnancy

Pregnancy affects asthma control in many women; approximately one-third of women report symptom worsening, one-third report symptom improvement, and one-third report no noticeable difference^10^. Poor maternal asthma control is associated with adverse outcomes, including increased risk of preterm birth, low birthweight, congenital malformations, perinatal death, and risk of childhood asthma^58–60^. In addition, a small reduction in the mother’s oxygen levels (e.g., during an asthma exacerbation) can result in severe, life-threatening fetal hypoxia^61^. It is, therefore, vital that pregnant women (and women who are thinking of becoming pregnant) are educated on taking their asthma medications as prescribed and have a plan for managing exacerbations^62^.

However, understandably, many women report being apprehensive about using asthma medication during pregnancy over concerns of teratogenicity, meaning that adherence to medications may decrease^63^. While the safety of most asthma medications (e.g., ICS, SABAs, long-acting beta-agonists, leukotriene receptor antagonists, OCS, and biologics) has not been unequivocally proven in pregnancy, they have now been used successfully for decades. Overall, evidence indicates asthma medications are safe in pregnancy, and their use is justified, as the benefits of good symptom control markedly outweigh the potential risks to mother and baby^10,64,65^.

Managing asthma symptoms during labor and delivery is also important, and guidelines advise women to continue with their usual asthma medications during this time^10^. Asthma symptoms occur in ~10% of deliveries^61^, and a cesarean section may be required if an acute exacerbation occurs^66^. Neonatal hypoglycemia is also a risk, especially if the woman takes high doses of beta-agonists in the 48 h before birth or if the baby is premature^10^. Oxytocin is the preferred drug to induce labor where necessary^67^. However, although this is a rare event, there is evidence from a small number of case studies that oxytocin can cause anaphylaxis in women with asthma^68^.

Author guidance:

- Inform pregnant patients of the detrimental effects of poorly controlled maternal asthma for their baby, both during pregnancy and after.
- Validate concerns using motivational communication and encourage pregnant patients to keep taking their asthma medication(s) as usual. The benefits of good symptom control outweigh the risks.
- As asthma symptoms may worsen or improve during pregnancy, instruct pregnant women on how to adjust their medication(s) appropriately and have an action plan for the management of exacerbations during pregnancy and labor.
- Inform pregnant women that although exacerbations during labor are rare, a cesarean section may be required in some instances if an exacerbation occurs^66^.
- Aspects of pregnancy can mimic asthma symptoms (e.g., breathlessness). Understanding differential diagnosis and how to assess it (e.g. using spirometry^67^), could be useful when treating pregnant women with asthma.

## Differential diagnoses

There may be difficulties in differentiating between symptoms caused by asthma and symptoms caused by other respiratory conditions (Table 1^20^^,42^^,43^^,69–79^). For example, asthma and COPD share common features which make differentiating them complicated, especially in older adults and smokers^80^. In developed countries, the prevalence of COPD in men and women is similar^81^. However, potentially due to HCP implicit bias and lack of spirometry use, women are more likely to receive a diagnosis of asthma rather than COPD^45,82^. Additionally, because anxiety disorders (e.g., panic disorders, which may be associated with hyperventilation and dysfunctional breathing) and obesity are more commonly diagnosed in women than men^42,43,69^, women who report respiratory symptoms may have their symptoms attributed to anxiety or obesity, rather than taken seriously and investigated further.

**Table 1:** Differential diagnoses of asthma in men and women.

More common in women	More common in men
Obesity^42,43^	COPD^43^ ^a^
Dysfunctional breathing, including vocal cord dysfunction and exercise-induced laryngeal obstruction (EILO)^69,70^	Lung cancer^71^ ^a^
Anxiety^43^	Idiopathic pulmonary fibrosis^72^
Bronchiectasis^73^	Heart failure^74^
Gastro-esophageal reflux^42,43^	Tuberculosis^75^
Upper airway cough syndrome^76^	
Pulmonary embolism^77^	
Systemic sclerosis-associated interstitial lung disease^78^	

*COPD* chronic obstructive pulmonary disease.

^a^COPD and lung cancer are generally higher in men globally, although incidence appears to be increasing in women, possibly due to increased tobacco use by women^71,81^, and increased susceptibility to nicotine^20^.

Author guidance:

- Determine whether symptoms are due to asthma or another condition and assess the severity of symptoms in all patients by using objective tests such as spirometry.
- Be cautious about inferring anxiety disorders or obesity as a cause of symptoms based on the patient’s sex or gender.

## Comorbidities

Comorbidities are associated with poor asthma outcomes^43^. Compared with men, women with asthma are more likely to have comorbidities, including obesity, osteoporosis, anxiety, and depression^43^. In addition, regular or frequent intake of OCS (and possibly high doses of ICS) to manage asthma symptoms increases the risk of developing side effects such as osteoporosis and cataracts^83^. As women are more likely than men to be prescribed OCS to manage their asthma symptoms^84^ (possibly because women report more severe symptoms than men^4^), they may be more at risk of these side effects. However, it is worth noting that corticosteroid use increases the risk of these comorbidities (including osteoporosis) in men as well as women^85^. Therefore, identifying patients who overuse corticosteroids is crucial for minimizing steroid-related comorbidities^86^.

Author guidance:

- Be aware that not all symptoms that occur in someone with asthma are due to their asthma. Careful discussions with the patient and objective tests to explore the distinguishing characteristics may be needed to avoid over-treatment with SABAs.
- Monitor both men and women for adverse events and comorbidities, including osteoporosis, particularly when using OCS as part of their asthma management regimen.

## Sex hormones

Some women with asthma find that their symptoms worsen during certain phases in their menstrual cycle, during pregnancy, and at menopause^10,87,88^. Menopause is also associated with an increased risk of new-onset asthma^89^. Certain hormonal contraceptives have been shown to improve asthma symptoms and decrease the risk of asthma in pre-menopausal women^90^. It is interesting to note that, while estrogen and progesterone are involved in asthma pathogenesis, testosterone may protect against inflammatory processes that cause asthma^7^. However, there is a paucity of research into whether testosterone replacement therapy has beneficial effects on asthma, or if low testosterone (e.g., during andropause) affects asthma in men^91^.

Author guidance:

- Recognize that some women with asthma may experience worse symptoms around menstruation, pregnancy and menopause.
- Hormonal contraceptives have the potential to improve asthma control in women whose symptoms fluctuate with their menstrual cycle.
- Fully assess women during pregnancy and at menopause who develop chest tightness or dyspnea to determine whether these symptoms are due to new-onset asthma, poor adherence to current asthma medication or other factors.

## Sex/gender-specific phenotypes

Neutrophilic, obese asthma is a distinct phenotype that is more common in women than men and is often difficult to manage^92,93^. Women with neutrophilic, obese asthma tend to have lower lung function and a poor response to corticosteroids compared with non-obese women; this is not the case with men who have this phenotype^93,94^. Women may be more likely to have neutrophilia than men due to body composition. Women tend to have more subcutaneous than abdominal adipose fat, which secretes more leptin (a pro-inflammatory mediator that recruits neutrophils to the airways^95^), leading to increased neutrophilic inflammation^96^. Identifying a patient’s phenotype is useful when ascertaining the most effective medication to prescribe. More recently, ‘treatable traits’ have been proposed for the management of complex airway diseases. These are phenotypic or endotypic characteristics that are clinically relevant, measurable, and treatable^97^.

Author guidance:

- Be wary of putting patients into ‘boxes’, and view asthma in terms of ‘treatable traits’ for which there are evidence-based interventions. With personalized medicine in mind, be adaptable to each patient’s individual characteristics.

## Discussion and conclusion

**Fig. 3:**
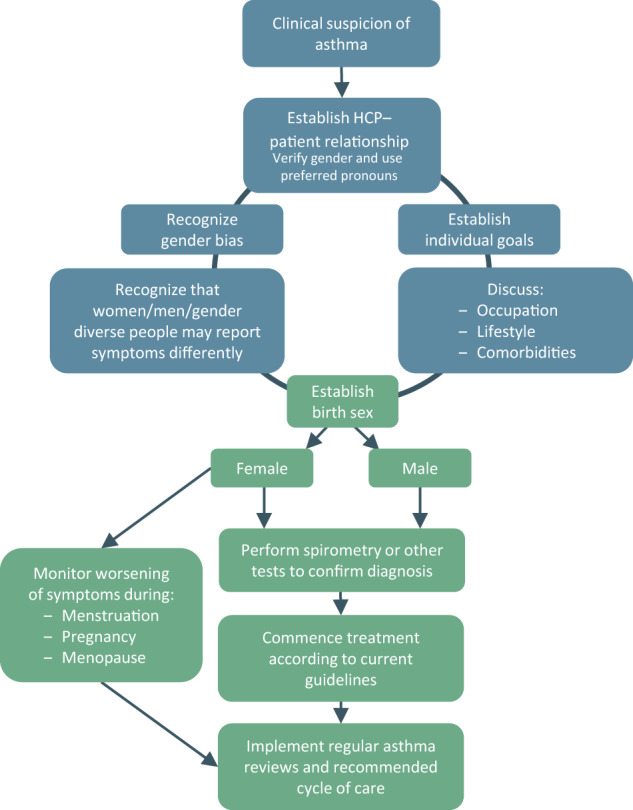
Considerations of sex and gender differences in patient asthma management. HCP healthcare professional.

**Fig. 4:**
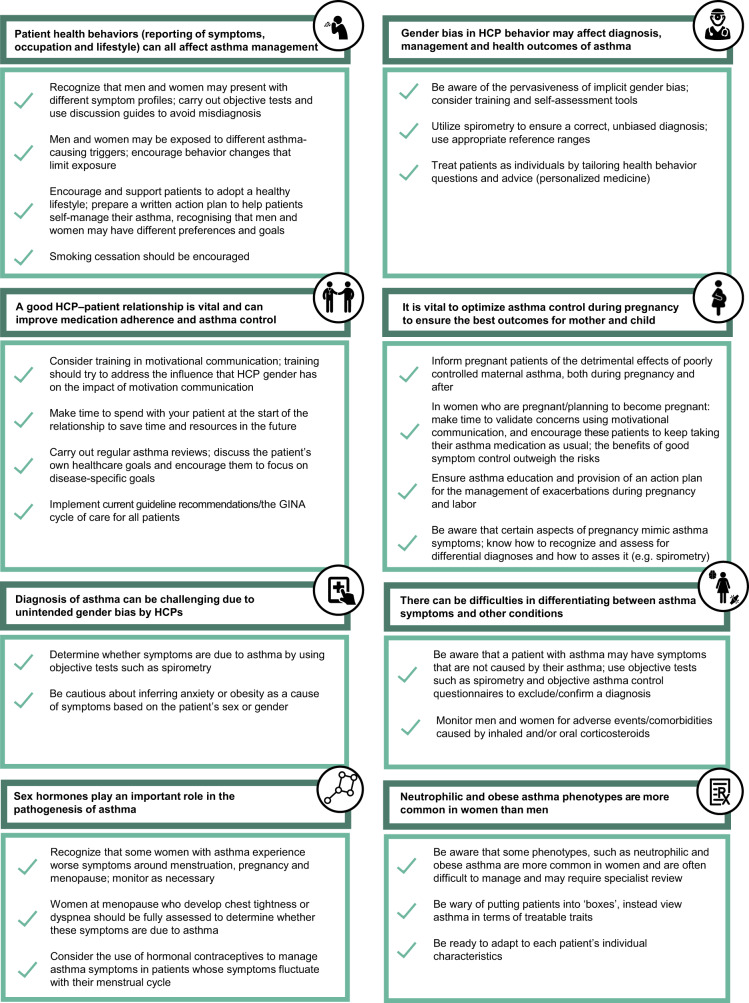
Summary: Suggested actions to minimize the impact of sex and gender differences in asthma diagnosis and management. GINA Global Initiative for Asthma, HCP healthcare professional.

Sex and gender can affect patient health behaviors, and implicit gender bias by HCPs is widespread and may affect diagnosis, management, and health outcomes^38^. Our suggestions should support HCPs to provide personalized asthma care for all patients, regardless of sex or gender. Figure 3 is a simple algorithm for navigating the considerations surrounding sex and gender differences when diagnosing and managing asthma. Figure 4 provides suggestions for minimizing the impact of sex and gender differences in asthma diagnosis and management.

There are now many gender terms that people can use for self-identification. In everyday practice, HCPs will increasingly start to see patients with complex gender identities—so, although it is important to keep sex/gender differences in mind while diagnosing/managing asthma, it is crucial that HCPs treat patients as individuals and strive to provide personalized asthma care for all patients, regardless of sex or gender.

In addition, good collaboration must exist between all HCPs involved in the management of each patient (PCP, pneumologist, gynecologist etc.). Further research would allow HCPs to better account for sex/gender in diagnosing and treating their patients with asthma. Urgent research is needed to investigate the links between hormone changes and asthma in women and men, and the effects of testosterone. To this end, there is a need for greater consideration of sex and gender in the design and analysis of clinical trials. Clarity regarding the use and definition of ‘male/female’ and ‘women/men’ is also necessary and would be a good starting point. Ultimately, knowledge of the causes of sex and gender disparities in asthma diagnosis and management should be a high priority for new research on how to increase gender equity and improve quality in clinical practice. In view of the evidence that sex- and gender-related differences and biases can significantly and adversely impact diagnosis and management if not recognized, it is concerning that these differences are largely not taught during HCP training, including in curricula, or discussed in guidance followed by HCPs^10,98^. It is, therefore, our opinion, that updated guidance and resources are urgently needed to help HCPs minimize the impact that sex and gender have on asthma diagnosis and management. For individualized asthma management to become part of normal HCP practice, it is essential that a new approach to asthma research, diagnosis, and management is taken, one that considers sex and gender, while treating the patient as an individual.

## Data Availability

This is a narrative review manuscript and does not report original research/data; thus, data sharing is not applicable.
